# Local alternating heat and cold stimulation affects hemodynamics and oxygenation in fatigued muscle tissue and autonomic nervous activity: a single-arm interventional study

**DOI:** 10.1186/s40101-024-00358-3

**Published:** 2024-03-25

**Authors:** Tomonori Sawada, Hiroki Okawara, Daisuke Nakashima, Kentaro Aoki, Mira Namba, Shuhei Iwabuchi, Yoshinori Katsumata, Masaya Nakamura, Takeo Nagura

**Affiliations:** 1 Diagnosis and Treatment Division, Nagura Orthopedic Clinic, Chuo, Tokyo, Japan; 2 Department of Orthopaedic Surgery, Keio University School of Medicine, Shinjuku, Tokyo Japan; 3 Department of Clinical Biomechanics, Keio University School of Medicine, Shinjuku, Tokyo, Japan; 4 Institute for Integrated Sports Medicine, Keio University School of Medicine, Shinjuku, Tokyo Japan; 5 Department of Cardiology, Keio University School of Medicine, Shinjuku, Tokyo Japan

**Keywords:** Alternating heat and cold stimulation, Trapezius muscle, Muscle hardness, Near-infrared spectroscopy, Intramuscular hemodynamics, Heart rate variability

## Abstract

**Background:**

Local alternating heat and cold stimulation as an alternative to contrast bath may cause intermittent vasoconstriction and vasodilation, inducing a vascular pumping effect and consequently promoting increased tissue blood flow and oxygenation. This study aimed to examine the effects of local alternating heat and cold stimulation, using a wearable thermal device, on the hemodynamics of fatigued muscle tissue and autonomic nervous activity.

**Methods:**

Twenty healthy individuals experienced fatigue in the periarticular muscles of the shoulder joint due to a typing task. Local alternating heat and cold stimulations were then applied to the upper trapezius muscle. Muscle hardness was measured using a muscle hardness meter, and muscle tissue hemodynamics and oxygenation were evaluated using near-infrared spectroscopy before and after the stimulation. Autonomic nervous activity was also evaluated using heart rate variability.

**Results:**

Alternating heat and cold stimulation decreased muscle hardness of the fatigued trapezius muscle from 1.38 ± 0.15 to 1.31 ± 0.14 N (*P* < 0.01). The concentration of total hemoglobin in the trapezius muscle tissue increased from − 0.21 ± 1.36 to 2.29 ± 3.42 µmol/l (*P* < 0.01), and the tissue hemoglobin oxygen saturation also increased from 70.1 ± 5.4 to 71.1 ± 6.0% (*P* < 0.05). Additionally, the heart rate variability parameter, which is an index of sympathetic nervous activity, increased from 3.82 ± 2.96 to 6.86 ± 3.49 (*P* < 0.01). A correlation was found between increased tissue hemoglobin oxygen saturation and increased parameters of sympathetic nervous activity (*r* = 0.50, *P* < 0.05).

**Conclusions:**

Local alternating heat and cold stimulation affected the hemodynamic response in fatigued muscle tissue and autonomic nervous activity. This stimulation is more efficient than conventional contrast baths in terms of mobility and temperature control and has potential as a new versatile therapeutic intervention for muscle fatigue.

**Trial registration:**

UMIN-CTR (UMIN000040087: registered on April 7, 2020, https://upload.umin.ac.jp/cgi-open-bin/ctr_e/ctr_view.cgi?recptno=R000045710. UMIN000040620: registered on June 1, 2020, https://upload.umin.ac.jp/cgi-open-bin/ctr_e/ctr_view.cgi?recptno=R000046359).

## Background

A contrast bath is a bathing method of alternating application of hot and cold water. It is believed to induce a vascular pumping effect by causing intermittent vasoconstriction and vasodilation, resulting in increased tissue blood flow and oxygenation, which improves healing, enhances tissue waste-product transportation to reduce edema, improves limb function, and promotes quicker recovery [1]. It is also expected to provide a relaxation effect and is widely used for recovery from fatigue after exercise, especially in athletes [2, 3]. However, contrast bath has limitations, such as the need for a large bath, difficulty with water temperature control (as it changes with each immersion), and hygiene issues (when multiple people use the same bath). Moreover, clear evidence is yet to be established, given that the temperature settings, number of treatments, and durations of hot and cold water application vary from one study to another [4–7].

Recently, a miniature apparatus with embedded Peltier elements was developed, and it allows easy program control and protocol assembly for heat and cold stimulation using a smartphone application. This small wearable device has the significant advantage of overcoming the problems of conventional contrast bath therapy, which is limited by mobility, location, water temperature control, and hygiene issues, as it can provide specific, rapid, and localized heating and cooling stimulation at increments of 0.1 °C. The device also enables optimal temperature protocol management to achieve its effect on the targeted muscle while preventing complications such as hot/cold burns. An additional advantage is the potential for more accurate verification of the effects of temperature change. In previous studies, we examined the effects of local alternating heat and cold stimulation using this device on a fatigued trapezius muscle and demonstrated that the stimulation improved muscle hardness and subjective fatigue [8, 9]. Muscles generally become harder under pathological conditions such as muscular damage, spasms, cramps, and edema [10–12]. Therefore, evaluation of muscle hardness is considered useful for assessing muscle fatigue associated with sustained muscle contraction. On the other hand, muscle hardness is measured from the body surface according to pressure and is constantly influenced by the skin, subcutaneous adipose tissue, muscles located below the target muscle, and bone [11, 13], making it inadequate for the evaluation of the effect of the stimulation on the target muscle tissue itself. Near-infrared spectroscopy (NIRS) is a widely used research instrument for measuring hemodynamics and oxygenation in muscle tissue noninvasively, and this technique may be useful for assessing how local alternating heat and cold stimulation alters the hemodynamics of fatigued muscle tissue due to sustained muscle contraction. Although previous studies have reported that hot pack therapy improves total hemoglobin (corresponding to blood volume) and oxygenation levels of a target muscle and tendon [14, 15], no study has examined the relationship between these improvements and changes in muscle hardness. Furthermore, autonomic nervous activity may also be involved in controlling the local circulation in skeletal muscles. The sympathetic nervous system plays a crucial role in mediating acute cardiovascular responses to stress. During exercise, the skeletal muscle uses a large amount of energy, which requires increased blood volume for oxygen consumption, and it is suggested to be involved in sympathetic cholinergic vasodilation in the skeletal muscle [16, 17]. However, no study has simultaneously evaluated the effects of contrast bath therapy and local alternating heat and cold stimulation on the local hemodynamics of skeletal muscle tissue and autonomic nervous activity and investigated the relationship between those effects. 

Accordingly, this study aimed to examine the effects of local alternating heat and cold stimulation on fatigue-induced upper trapezius muscle tissue hemodynamics and autonomic nervous activity, in addition to previous muscle hardness measurements. We hypothesized that alternating heat and cold stimulation not only reduces muscle hardness but also increases blood volume and oxygenation in the muscle tissue and sympathetic nervous activity for vasodilation in the skeletal muscle, and some correlation would exist between these changes. To date, the relationship between increased blood volume in targeted fatigued muscle and autonomic nervous activity due to the use of contrast baths is not well understood. Therefore, the findings of this study may provide support for the physiological effects of contrast baths and suggest that local alternating heat and cold stimulation could be a potentially beneficial approach for muscle fatigue as an alternative to conventional contrast baths.

## Methods

### Study design and participants

This was a non-randomized, single-arm interventional study registered with the UMIN Clinical Trials Registry (registration numbers UMIN000040087 and UMIN000040620). In Japan, shoulder stiffness is the second most common complaint (57.2 per 1000 population) after back pain among men and is the most frequently occurring condition (113.8 per 1000 population) among women [18]. Moreover, since it is believed that complaints of shoulder stiffness among young adults are due to prolonged smartphone use in recent years, this study recruited mainly young men and women. After recruiting participants from one clinic and one university between March 2021 and August 2022, 20 healthy young individuals (males, 13; females, 7) without any orthopedic abnormalities of the neck or shoulders were included in this study (Table 1). All the participants were informed of the purpose of the study. Those who refused to participate in the study or did not provide informed consent were excluded. The participants’ average daily smartphone use and average typing time over the past month were recorded using a questionnaire.

**Table 1:** Participant characteristics

	All (*n* = 20)	Males (*n* = 13)	Females (*n* = 7)
Age (years)	21.4 ± 3.2	21.6 ± 3.9	20.9 ± 1.0
Height (cm)	169.4 ± 7.4	173.8 ± 5.0	161.3 ± 3.2
Weight (kg)	64.5 ± 15.3	72.1 ± 13.8	50.4 ± 3.5
Body mass index (kg/m^2^)	22.3 ± 4.2	23.9 ± 4.4	19.4 ± 0.8
Dominant hand (n)			
Right	19	13	6
Left	1	0	1
Time of smartphone use per day (h)	4.9 ± 2.1	5.7 ± 2.1	3.4 ± 1.2
Time of typing per day (h)	1.0 ± 1.6	1.2 ± 2.0	0.6 ± 0.3

### Experimental protocol

All the participants were asked to visit our laboratory where the room temperature was set between 24 and 26 °C to ensure that the experiment was conducted in a similar environment as much as possible. The participants performed the typing task at a desk for 15 min using a laptop computer to induce fatigue around the shoulder, according to previous studies [9, 19]. The participants were instructed to maintain the same posture while transcribing as much text as possible into the document entry software. The text used in the typing task was an out-of-copyright Japanese novel. Intervention with alternating heat and cold stimulation was implemented using a commercially available wearable thermal device (WTD) (REON POCKET 2; Sony Group Corporation, Tokyo, Japan). The WTD contains a Peltier element that uses voltage regulation to produce surface heating or cooling in an area of 4.5 × 5.5 cm. It can be operated using a smartphone application to provide repeated cooling, heating, and pausing for a fixed number of seconds, and the intensity can be adjusted in four levels from 1 to 4 for the heating and cooling stimulations separately, with level 1 as the weakest and level 4 as the strongest. The intensity of the heat stimulation was set at level 3, and that of the cold stimulation was set at level 4. The WTD was taped over the skin of the upper trapezius muscle on the dominant side, and the center of the device was 2 cm lateral to the midpoint between the 7th cervical spinous process and the tip of the acromion [9, 20, 21]. Heat stimulation was applied for 3 min, followed by cold stimulation for 1 min, for a total of five sets. Additionally, 10 s of movement cessation was allowed between the heat and cold stimulations to reduce thermal stress on the WTD. During the stimulation, the participants rested in a relaxed position on a chair with a backrest. In this study, the following parameters were evaluated before and after the alternating heat and cold stimulation: trapezius muscle hardness using a muscle hardness meter, trapezius muscle tissue hemodynamics and oxygenation using NIRS, and autonomic nervous activity using heart rate variability (HRV).

### Measurements

Muscle hardness was quantitatively evaluated by a trained examiner using a portable muscle hardness meter (NEUTONE TDM-Z2; TRY-ALL Corp., Chiba, Japan). A similar device was used in previous studies [22–24]. Our previous study showed that the muscle hardness meter had excellent intratester reliability for the trapezius muscle (*ICC*_1,5_ = 0.992–0.995) [25]. This meter displays values on a scale of 0 to 100 without units. We converted the scale values to Newton (N) using the following formula, based on the manufacturer’s recommendations: *N* = 0.023 × measured value + 0.532. The measurement point was 2 cm lateral to the midpoint between the 7th cervical spinous process and the tip of the acromion as well as the center of the WTD attachment. Measurements were obtained five times at each time point, and the mean value was used for analysis. Muscle hardness was not only evaluated before and after the alternating heat and cold stimulation intervention but also at baseline before the typing task to confirm whether the task induced muscle fatigue and increased muscle hardness, as observed in our previous study [9].

Muscle tissue hemodynamics and oxygenation of the upper trapezius were evaluated using an NIRS system (NIRO-200NX; Hamamatsu Photonics K.K., Hamamatsu, Japan) that uses the modified Beer-Lambert (MBL) method and spatially resolved spectroscopy (SRS). A pair of photoemission and photodetection probes were set at a constant distance of 4 cm in a specialized rubber holder, which was fixed to the skin of the muscle belly adjacent to the alternating heat and cold stimulation area using a double-sided adhesive sheet. Changes in the concentration of oxyhemoglobin (ΔOxyHb), deoxyhemoglobin (ΔDeoxyHb), and total hemoglobin (ΔTotalHb) [ΔoxyHb + ΔDeoxyHb], which is an index of the local blood volume [26, 27], were measured using MBL methods. Additionally, tissue hemoglobin oxygen saturation (tissue oxygenation index, TOI, expressed in percentage) was calculated using SRS [28–30]. The data for these parameters were continuously obtained at 1 Hz. Among the obtained data, the average values 2 min before and after the intervention were used for analysis.

HRV analysis was performed using dedicated analysis software (Reflex Meijin; Crosswell Corp., Yokohama, Japan). The R-R interval, which is the interval between one QRS wave and the next, was collected at 1000 Hz using a heart rate monitor (MWT-001; AHM Electronics Co., Ltd., Kodaira, Japan) attached to the chest. Two power spectrum components, a high-frequency (HF) component (0.15–0.4 Hz) and a low-frequency (LF) component (0.04–0.15 Hz), were continuously calculated using the maximum entropy method while shifting every beat for a 30-s period of HRV [31]. HF components can be used as a general index of parasympathetic nervous activity, and the LF/HF ratio can be used as an index of sympathetic nervous activity [32, 33]. In addition to HF, LF, and LF/HF, the R-R interval coefficient of variation (CVRR [%] = standard deviation/mean of the R-R interval during the previous 30 s × 100) was also measured. Among the obtained data, the average values 2 min before and after the intervention were used for analysis.

### Statistical analysis

Normality of the data distribution was assessed using the Shapiro–Wilk test. Initially, the values of each parameter before and after the intervention were compared using paired *t*-tests. Regarding muscle hardness, the baseline values before the typing task were further compared with the values before the intervention (after typing) using paired *t*-tests. In addition, to assess the possibility of differences in muscle hardness values by sex, baseline values were compared between males and females using unpaired *t*-tests. The effect size for each comparison was calculated based on Cohen’s *d* [34]. Moreover, to examine the association of the intervention with changes in muscle stiffness, NIRS, and HRV parameters, correlations between the changes in each parameter (post-intervention value minus pre-intervention value) were assessed using Pearson’s or Spearman’s correlation coefficient, based on the normality distribution of the parameter data. All statistical analyses were performed using SPSS version 25.0 (IBM Corp., Armonk, NY, USA), with statistical significance set at 0.05.

## Results

### Comparison of muscle hardness before and after the fatigue typing task and differences in muscle hardness between sexes

The 15-min typing task significantly increased muscle hardness after the task compared with that before the task (1.34 ± 0.15 N vs. 1.38 ± 0.15 N, *P* < 0.05, *d* = 0.56). No significant differences in muscle hardness before the typing task were found between sexes (male: 1.32 ± 0.15 N, female: 1.40 ± 0.15 N, *P* = 0.30, *d* = 0.50).

### Comparison of muscle hardness, NIRS, and HRV parameters before and after the intervention

**Fig. 1:**
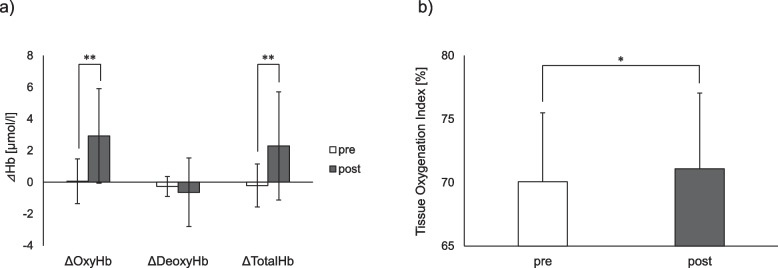
Mean values in muscle tissue hemodynamics and oxygenation indices using NIRS at pre- and post-intervention. **a** Hemoglobin concentration changes (ΔOxyHb, ΔDeoxyHb, and ΔTotalHb) by MBL. **b** TOI by spatially resolved spectroscopy. *P < 0.05 and **P < 0.01. Error bars indicate standard deviation. NIRS, near-infrared spectroscopy; OxyHB; oxyhemoglobin; DeoxyHB, deoxyhemoglobin; TotalHb, total hemoglobin; MBL, modified Beer-Lambert; TOI, tissue oxygenation index

**Fig. 2:**
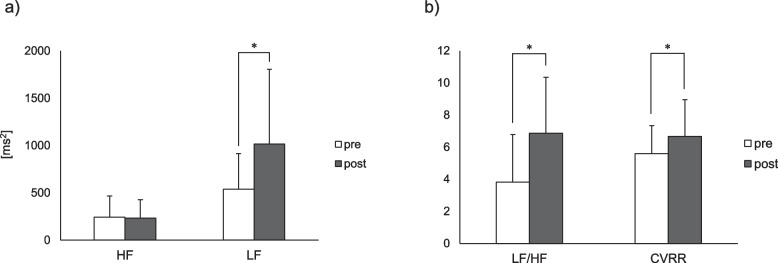
Mean values of HRV parameters at pre- and post-intervention. **a** HF and LF. **b** LF/HF and CVRR. *P < 0.01. Error bars indicate standard deviation. HRV, heart rate variability; HF, high frequency; LF, low frequency; CVRR, coefficient of variation of R-R interval

Muscle hardness was significantly reduced after the intervention compared with that before the intervention (1.38 ± 0.15 N vs. 1.31 ± 0.14 N, *P* < 0.01, *d* = 0.97), suggesting that the intervention caused soft tissue softness, including muscle palpable from the surface of the body. Figure 1 presents the mean values of the muscle tissue hemodynamics and oxygenation indices using NIRS. OxyHb, ΔTotalHb, and TOI increased after the intervention (0.06 ± 1.40 µmol/l vs. 2.92 ± 2.99 µmol/l, *P* < 0.01, *d* = 1.15; − 0.21 ± 1.36 µmol/l vs. 2.29 ± 3.42 µmol/l, *P* < 0.01, *d* = 0.95; and 70.1 ± 5.4% vs. 71.1 ± 6.0%, *P* < 0.05, *d* = 0.17, respectively). These results indicate that the intervention increased blood perfusion, including oxyhemoglobin, into the targeted trapezius muscle tissue and facilitated oxygenation. The mean values of the HRV-related indices are also shown in Fig. 2. The LF, LF/HF, and CVRR increased significantly after the intervention (538.6 ± 375.3 ms^2^ vs. 1017.2 ± 789.4 ms^2^, *P* < 0.01, *d* = 0.59; 3.82 ± 2.96 vs. 6.86 ± 3.49, *P* < 0.01, *d* = 0.91; and 5.59 ± 1.75% vs. 6.66 ± 2.30%, *P* < 0.01, *d* = 0.49, respectively), whereas no statistically significant difference was observed in the HF before and after the intervention. These HRV-related results imply that the alternating heat and cold stimulation increased sympathetic nervous activity.

### Relationship between change in muscle hardness and changes in the other parameters by intervention

The correlation between changes in muscle hardness and changes in NIRS or HRV parameters after intervention with alternating heat and cold stimulation was investigated. No significant correlation with changes in muscle hardness was found for the NIRS- or HRV-related outcomes (Table 2).

**Table 2:** Correlations between change in muscle hardness and changes in other parameters

	Muscle hardness	
	Spearman *r*	*p*-value
ΔOxyHb	− 0.124	0.602
ΔDeoxyHb	− 0.119	0.17
ΔTotalHb	− 0.095	0.691
TOI	0.365	0.114
HF	− 0.391	0.088
LF	0.012	0.960
LF/HF	0.273	0.245
CVRR	0.054	0.820

Correlation analysis was performed between the changes in each participant’s post- and pre-intervention values

*OxyHB* oxyhemoglobin, *DeoxyHB* deoxyhemoglobin, *TotalHb* total hemoglobin, *TOI* tissue oxygenation index, *HF* high frequency, *LF* low frequency, *CVRR* coefficient of variation of R-R interval

### Relationship between changes in NIRS and HRV parameters by intervention

**Fig. 3:**
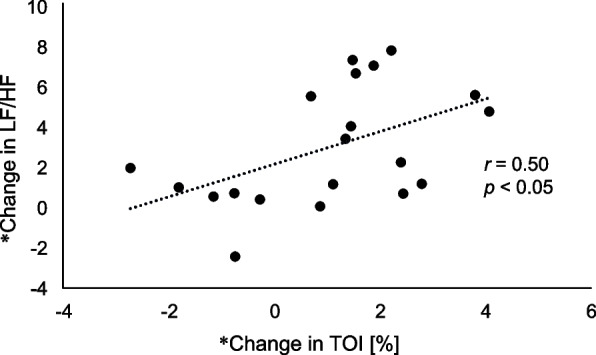
Scatter diagram representing change in TOI and change in LF/HF. *Value was calculated as the pre-intervention value minus the post-intervention value. TOI, tissue oxygenation index; LF, low frequency; HF, high frequency

The correlations between changes in NIRS and HRV outcomes after the intervention are shown in Table 3. Among them, the change in TOI was positively correlated with the change in the LF/HF ratio (*r* = 0.50, *P* < 0.05) (Fig. 3).

**Table 3:** Correlations between changes in NIRS and HRV parameters

	ΔOxyHb		ΔDeoxyHb		ΔTotalHb		TOI	
	Correlation coefficient	*p*-value	Correlation coefficient	*p*-value	Correlation coefficient	*p*-value	Correlation coefficient	*p*-value
HF	0.02a	0.79	0.084a	0.724	0.015a	0.950	− 0.308a	0.186
LF	0.177a	0.454	− 0.041a	0.85	− 0.093a	0.696	0.116a	0.627
LF/HF	0.309b	0.185	− 0.322a	0.166	0.088b	0.713	0.499b	0.025
CVRR	0.252b	0.283	− 0.260a	0.268	0.124b	0.603	0.375b	0.103

Correlation analysis was performed between the changes in each participant’s post-intervention and pre-intervention values

*NIRS* near-infrared spectroscopy, *HRV* heart rate variability, *OxyHB* oxyhemoglobin, *DeoxyHB* deoxyhemoglobin, *TotalHb* total hemoglobin, *TOI* tissue oxygenation index, *HF* high frequency, *LF* low frequency, *CVRR* coefficient of variation of R-R interval

^a^Spearman’s correlation coefficient

^b^Pearson’s correlation coefficient

## Discussion

To the best of our knowledge, this is the first study to demonstrate that superficial local alternating heat and cold stimulation on fatigued muscle affects the hemodynamics of muscle tissue and autonomic nervous activity. Our results showed that alternating heat and cold stimulation improved muscle hardness, which is consistent with previous reports [8, 9]. However, no relationship was found between changes in muscle hardness and changes in hemodynamics or autonomic nervous activity. The alternating heat and cold stimulation increased ΔTotalHb with an increase in ΔOxyHb and TOI. In addition, the results of the HRV analysis showed an increase in LF/HF, suggesting an increase in sympathetic nervous activity. Furthermore, a significant correlation was observed between the changes in TOI and the LF/HF ratio induced by alternating warm and cold stimulation, suggesting that sympathetic nervous activity is involved in muscle tissue oxygenation.

Our results indicate that the improvement in muscle hardness by alternating heat and cold stimulation failed to reflect changes in hemodynamics in the targeted muscle tissue and autonomic nervous activity. That is, the findings suggest that changes in muscle hardness may be influenced by physiological mechanisms different from muscle tissue hemodynamics and autonomic nervous activity. Muscle hardness is defined as the resistance of the muscle tissue to perpendicular pressure [11, 35]. Thermotherapy is generally expected to increase tissue temperature, local blood flow, and soft tissue extensibility, cause local vasodilation, increase metabolite production, and reduce muscle spasms [36, 37]. For the present alternating stimulation, similar physiological effects are expected to occur because heat is the most prevalent stimulation. Muscle hardness measured using a push-in muscle hardness meter is believed to specifically assess soft tissue extensibility in these physiological effects, but muscle hardness values also include the effects of the skin, subcutaneous tissue, and deep muscle layers [11, 13]. Previous studies have evaluated trapezius muscle stiffness in healthy young volunteers using a muscle hardness meter and ultrasound elastography and showed no correlation between the obtained values [23, 25]. Thus, although alternating heat and cold stimulation resulted in a decrease in muscle hardness value, these changes may reflect changes in the extensibility of not only the target muscle but also the surrounding soft tissues. The improvement in peripheral blood flow expected from superficial heat stimulation is generally scarce in muscles located deeper in the skin and the subcutaneous tissue [37]. Therefore, changes in muscle hardness value might be more reflective of changes in the extensibility of the superficial soft tissue including the skin and subcutaneous tissue or blood flow within the targeted muscle tissue. Nevertheless, muscle hardness can easily be measured in clinical practice using a muscle hardness meter because it is similar to palpation, and the equipment is relatively inexpensive and portable. Therefore, further research to scientifically identify the factors that contribute to changes in these values would be of great clinical importance.

In this study, intramuscular hemodynamics were evaluated using NIRS to detect physiological changes in the target muscle tissue due to alternating heat and cold stimulation. The present study found that local stimulation of the upper trapezius muscle increased intramuscular oxygenated blood volume, which is consistent with findings in a previous study that evaluated intramuscular hemodynamics during contrast baths to the lower leg muscles using NIRS [38]. Generally, heat causes vasodilation and increases blood flow [36]. In contrast, superficial heat agents produce more pronounced vasodilation in the local cutaneous blood vessels but less vasodilation in deeper vessels that run through the muscles [37]. Hence, at first glance, the WTD used in this study is presumed to have a limited effect because the stimulation area is narrower (4.5 × 5.5 cm) than that of conventional contrast bath therapy. However, the results showed increased vasodilation and oxygen supply to the target muscle tissue. Desk workers engaged in intensive and prolonged computer use are known to be at a higher risk of neck-to-shoulder pain [39–41]. Furthermore, with the recent explosion of smartphone use, musculoskeletal disorders of the neck have been increasing, especially among university students [42, 43]. Obstruction of blood flow and a reduction in muscle tissue oxygenation during sustained repetitive work have been suggested to contribute to the development of upper extremity muscle disorders [44]. In addition, muscle blood flow is believed to have a significant impact on the development of muscle fatigue [45] because of its important role in the delivery of metabolic waste products (e.g., inorganic phosphate, lactic acid, and H +) that contribute to muscle fatigue [46, 47]. Thus, the localized superficial alternating heat and cold stimulation using a WTD in this study overcomes the limitations of conventional alternating baths and may be an effective intervention or prevention strategy for desk workers and university students with symptoms in the neck and shoulder regions.

The results of this study demonstrated that local alternating heat and cold stimulation also affects HRV parameters, which is an index of sympathetic nervous activity. To date, no study has examined the effects of local alternating heat and cold stimulation or conventional contrast baths on autonomic nervous activity. Massage therapies for relaxation have been reported to produce beneficial physiological effects such as a decrease in heart rate and blood pressure [48, 49]. Decreased heart rate is believed to be caused by either increased parasympathetic nervous activity or decreased sympathetic nervous activity [50]. Several previous studies have shown that massage increases the HF component, which is an index of parasympathetic nervous activity [51], and decreases the LF/HF ratio, which is an index of sympathetic nervous activity [52, 53]. However, in this study, alternating heat and cold increased LF, LF/HF, and CVRR, suggesting that sympathetic nervous activity increased. More interestingly, the correlation analysis between changes in HRV and NIRS parameters revealed a significant positive correlation between LF/HF and TOI. Generally, the activation of the sympathetic nervous activity causes blood vessels to constrict and the heart rate to increase. Consequently, blood pressure and the amount of perfusion in peripheral tissues increase. Previous studies have suggested that sympathetic cholinergic vasodilation in skeletal muscles is involved in increased oxygen demand in muscle tissues due to increased exercise load [16, 17]. In addition, heat stress is also believed to induce important physiological responses similar to those induced by exercise [54]. These include increase in skin and muscle blood flow, heart rate, and sympathetic nervous activity [55, 56]. Sympathetic nervous activity increases by approximately 90% in the muscle vasculature and 300–600% in the skin vasculature during passive heat stress [56–58]. Further, detailed investigation is needed to determine whether the sympathetic vasodilation effects in skeletal muscle can be induced by local alternating warm and cold stimulation as well as during intense exercise or whole-body heating. Nevertheless, the new finding of the correlation between increased sympathetic nervous activity and increased oxygenation in the muscle tissue by alternating heat and cold stimulation is an interesting result that could partly explain the stimulation effects. 

The limitations of this study warrant further discussion. First, because this study included only healthy young male and female individuals (age range, 19–35 years), the results cannot be generalized to older adults or patients with neck and shoulder pain. Second, this was a single-arm interventional study and did not perform comparisons with the no-stimulation condition or detailed examination of whether the effects of alternating heat and cold stimulation on muscle tissue hemodynamics and HRV differ between fatigued and non-fatigued conditions. To address these two limitations, future research should be conducted to examine whether similar effects can be obtained by expanding the intervention to include older adults and patients with neck and shoulder pain and investigate the effects of the alternating stimulation, eliminating temporal effects, by conducting an additional no stimulation condition. Third, the factor responsible for the changes in muscle hardness measured by the muscle hardness meter remains unclear. Future research could explain the changes in muscle hardness by assessing not only the targeted muscle tissue but also superficial skin blood flow or subcutaneous tissue stiffness. Finally, changes in autonomic nervous activity observed with local alternating heat and cold stimulation cannot be applied to the effects of conventional contrast bath therapy. A previous study examining the effects of warm hand bathing on autonomic nervous activity reported an increase in heart rate and a decrease in HF, resulting in increased sympathetic activity [59]. In contrast, other studies have demonstrated that warm footbaths decrease the LF/HF ratio and suppress sympathetic activity [60, 61]. Therefore, the effects on autonomic nervous activity may vary depending on the site and extent of stimulation.

## Conclusions

Similar to that in a contrast bath, the local alternating heat and cold stimulation used in this study improved hemodynamics and oxygenation indices in the target muscle tissue. The results of the HRV analysis indicated that sympathetic nervous activity increased by stimulation. Furthermore, an association was found between an increase in oxygenation indices in the muscle tissue and changes in sympathetic nervous activity parameters. These findings enhance our understanding of the physiological effects of contrast baths on muscle tissue hemodynamics. This stimulation is more efficient than conventional contrast baths in terms of mobility and temperature control and has potential as a new versatile therapeutic intervention for muscle fatigue.

## Data Availability

The datasets used and/or analyzed in the current study are available from the corresponding author upon reasonable request.

## Abbreviations

CVRR: Coefficient of variation of R-R interval
HF: High frequency
HRV: Heart rate variability
LF: Low frequency
MBL: Modified Beer-Lambert
NIRS: Near-infrared spectroscopy
SRS: Spatially resolved spectroscopy
TOI: Tissue oxygenation index
